# Molecular Analysis of Two Different MRSA Clones ST188 and ST3268 From Primates (*Macaca* spp.) in a United States Primate Center

**DOI:** 10.3389/fmicb.2018.02199

**Published:** 2018-10-09

**Authors:** Marilyn C. Roberts, Andrea T. Feßler, Stefan Monecke, Ralf Ehricht, David No, Stefan Schwarz

**Affiliations:** ^1^Department of Environmental and Occupational Health, University of Washington, Seattle, WA, United States; ^2^Institute of Microbiology and Epizootics, Centre for Infection Medicine, Department of Veterinary Medicine, Freie Universität Berlin, Berlin, Germany; ^3^Abbott (Alere Technologies GmbH), InfectoGnostics Research Campus Jena, Jena, Germany; ^4^Institut für Medizinische Mikrobiologie und Hygiene, Medizinische Fakultät “Carl Gustav Carus”, Dresden, Germany; ^5^Leibniz Institute of Photonic Technology (IPHT), Jena, Germany

**Keywords:** MRSA, *Macaca mulatta*, *Macaca fascicularis*, *Macaca nemestrina*, novel *spa* type, multi-drug resistance, colonization, infection

## Abstract

Methicillin-resistant *Staphylococcus aureus* (MRSA) were identified in macaques, their environmental facility, and nasal cultures of personnel from the Washington National Primate Research Center [WaNPRC] and included MRSA ST188 SCC*mec* IV and MRSA ST3268 SCC*mec* V. The aim of the current study was to determine the carriage of virulence genes, antibiotic resistance genes, and other characteristics of the primate MRSA isolates to determine if there were any obvious differences that would account for differences in transmission within the WaNPRC facility. In total, 1,199 samples from primates were tested for the presence of MRSA resulting in 158 MRSA-positive samples. Fifteen ST188 isolates (all from *Macaca nemestrina*) and nine ST3268 (four from *Macaca mulatta*, two from *Macaca fascicularis*, three from *M. nemestrina*), were selected for further characterization. All but one of the 15 ST188 isolates had *spa* type t189 and the remaining one had *spa* type t3887. These isolates were resistant to β-lactams [*blaZ, mecA*], macrolides/lincosamides [*erm*(B)], aminoglycosides [*aacA-aphD*], and fluoroquinolones. Five isolates were additionally resistant to tetracyclines [*tet*(K)] and had elevated MICs for benzalkonium chloride [*qacC*]. In comparison, the nine ST3268 isolates had the related *spa* types t15469 (*n* = 5) and t13638 (*n* = 4). All nine ST3268 isolates were resistant to β-lactams [*blaZ, mecA*], and tetracyclines [*tet*(K)]. Some isolates were additionally resistant to aminoglycosides [*aacA-aphD*], fluoroquinolones and/or showed elevated MICs for benzalkonium chloride [*qacC*]. In contrast to the ST188 isolates, the ST3268 isolates had the enterotoxin gene cluster *egc* [*seg, sei, selm, seln, selo, selu*] and enterotoxin genes *sec* and *sel*. The two clones have differences regarding their *spa* types, virulence and antibiotic resistance genes as well as ST and SCC*mec* types. However, the data presented does not provide insight into why ST188 spreads easily while ST3268 did not spread within the WaNPRC in-house primates.

## Introduction

Methicillin-resistant *Staphylococcus aureus* (MRSA) is an important opportunistic pathogen in human and veterinary medicine and can be a harmless colonizers but may also cause severe and live-threatening infections (Foster, 2017). MRSA consists of numerous pandemic, epidemic and sporadic clones (Monecke et al., 2011). There is very limited data on the carriage of *S. aureus* (including MRSA) in captive primates with even more limited data on MRSA carriage in wild primates in their natural habitats (Taylor and Grady, 1998; Weese, 2010; Hanley et al., 2012; Schaumburg et al., 2013; Soge et al., 2016; Roberts et al., 2018). Prior to 2014, neither *S. aureus* nor MRSA were identified in macaques from the Washington National Primate Research Center [WaNPRC], Seattle WA, United States. However, in 2014, there were nine cases of MRSA. This led to the 2015 carriage study, which determined that 17.6% of the macaques, 3.6% of the primate environmental facility samples and 2.5% of the primate personnel carried MRSA (Soge et al., 2016). Initially, all the isolates from macaques, environment and one of the personnel isolates were MRSA ST188 SCC*mec* IV [MLST profile 3, 1, 1, 8, 1, 1, 1, 1]. MRSA ST188 are not commonly found in North America^1^ (Soge et al., 2016). Our previous work showed that the ST188 SCC*mec* IV represented a clone and was easily transferred between macaques in the same cage, the same room or between playmates and contaminated the primate environment. One primate researcher carried MRSA ST188 SCC*mec* IV in the nose, while another carried a normally human isolated ST8 SCC*mec* IV (Soge et al., 2016).

In May 2015, a large shipment of macaques [> 90 *Macaca nemestrina*] from out-of-state, from other United States Primate Research Centers and arrived at WaNPRC. Most of these animals were colonized with MRSA ST3268 SCC*mec* V [MLST profile 1, 14, 430, 214, 10, 303, 329] (Soge et al., 2016). This was a novel sequence type (ST) and did not seem to readily spread within the WaNPRC until later in 2015 when four MRSA ST3268-positive animals were identified. These appeared to have been exposed and acquired ST3268 from a contaminated common procedure room within in the WaNPRC. These animals were also positive for the simian immunodeficiency virus (SIV) (Soge et al., 2016). Since the first introduction of MRSA ST3268, the WaNPRC has continued to receive MRSA ST3268-positive animals with new shipments of primates but no spread of this clone was observed. More recently, MRSA ST3268 isolates and a single locus variant MRSA ST2817 isolates have been detected in Singaporean long-tailed macaques (*Macaca fascicularis*) used in experimental surgery in 2014 and one person who worked in animal husbandry at the facility. These animals originated from Vietnam (Hsu et al., 2017). ST3268 differs by one housekeeping gene [*glp*] from ST2817, which has been identified in Asia.

The hypothesis of the current study was that there were some differences in the carriage of virulence factors, antibiotic resistance genes, and other characteristics between the two MRSA clones ST188 and ST3268 that might suggest why there is a different transmission frequency among the WaNPRC macaques.

## Materials and Methods

### Primate Sampling, MRSA Isolation and Verification

A total of 1,199 primate samples from the WaNPRC facility was tested for the presence of MRSA between May and August 2015. The animals [*M. fascicularis, Macaca mulatta*, and *M. nemestrina*] were in-house animals, as well as, out-of-state macaques shipped to the facility. The isolates were previously collected as part of the general care of the animals approved by the Institutional Animal Care and Use Committee at the University of Washington, United States, and the American Society of Primatologists (ASP) Principles for the Ethical Treatment of Nonhuman Primates (Soge et al., 2016). In addition, other animals were obtained from different commercial vendors and different sources outside the United States and were investigated shortly after their arrival at the WaNPRC during the quarantine period. MRSA-positive animals were given baths with chlorhexidine scrub for five consecutive days. The chlorhexidine was applied to the entire body and scrubbed with a surgical scrub brush with extra time spent cleaning axillary, perianal and preputial areas. In addition, animals received nasal application of mupirocin ointment 2% given twice daily for 5 days at the same time. Animals were sampled again at two and four weeks after initial MRSA positive culture and chlorhexidine and mupirocin treatment and retreated if still MRSA positive. All animals in the colony had initial nasals cultures done, while wound and/or skin infections were also sampled when present. All samples were taken from ketamine-sedated animals using standard microbiological swabs; BD BBL CultureSwab Plus Amies Medium (Becton Dickinson, Franklin Lakes, NJ, United States) and/or Starplex Starswab II (Starplex Scientific, Etobicoke, ON, Canada) as previously described (Roberts et al., 2011; Soge et al., 2016). For the current study, colonies were identified as *S. aureus* by production of β-hemolysis on blood agar plates and a positive Staphaurex^®^ test following manufacturer’s instructions (Remel, Lenexa, KS, United States; Soge et al., 2016). No isolate was selected unless they met these criteria (Soge et al., 2016). The presence of the alternative PBP2’ was determined with the Thermo Scientific PBP2’ latex agglutination test kit^®^ using instruction from the manufacturer (Thermo Fisher Scientific Remel Products, Lenexa, KS, United States). MRSA isolates were stored at -80°C. Isolates were selected without knowledge of the host primate species. This included 15 of 56 MRSA ST188 SCC*mec* IV isolates obtained from 36 animals and selected from various sample sites including animals that appeared refractory to mupirocin topical treatment. The 15 ST188 isolates came from ten *M. nemestrina* hosts and included three skin samples, and 12 nasal samples (**Table 1**). From *M. nemestrina* Z1242, three different nasal isolates Z1242N1, Z1242N2, Z1242N3, were selected taken on Feb 2, April 24, and June 5, 2015 to determine if the same strain was present over the 5 month time period. This animal was treated with chlorhexidine scrub and nasal application of mupirocin ointment between samplings. One *M. nemestrina* [Z121] had paired nasal Z121N and skin Z121S isolates taken May 29, 2015, while *M. nemestrina* Z123 had two isolates from two nasal samples [Z123N1 and Z123N2] isolated May 15 and 29, 2015 and a skin sample [Z123S] isolated May 29, 2015 (**Table 1**). This animal was treated with chlorhexidine scrub and mupirocin ointment when first identified as MRSA positive in May 2015. All these animals were from the WaNPRC facility.

**Table 1:** Characterizations of the MRSA ST188 SCC*mec* V and ST3268 SCC*mec* V.

Animal ID	MLST	Host	Date collected	Source	*spa* type	PFGE type (SmaI)	Resistance patterns	Resistance genes	Enterotoxins, leukocidins, other genes
A112	ST188	*M. nemestrina*	2/27/2015	Nasal	t189	A2	PEN, OXA, ERY, CLI, GEN, KAN, CIP	*blaZ, mecA, erm*(B), *aacA-aphD*	*hlgA/lukF/lukS, lukD/lukE, lukX/lukY*
K062	ST188	*M. nemestrina*	8/7/2015	Nasal	t189	A1	PEN, OXA, ERY, CLI, GEN, KAN, TET, CIP, BAC	*blaZ, mecA, erm*(B), *aacA-aphD, tet*(K), *qacC*	*hlgA/lukF/lukS, lukD/lukE, lukX/lukY*
L091	ST188	*M. nemestrina*	4/3/2015	Nasal	t189	A	PEN, OXA, ERY, CLI, GEN, KAN, CIP	*blaZ, mecA, erm*(B), *aacA-aphD*	*hlgA/lukF/lukS, lukD/lukE, lukX/lukY*
Z121N	ST188	*M. nemestrina*	5/29/2015	Nasal	t189	A	PEN, OXA, ERY, CLI, GEN, KAN, CIP	*blaZ, mecA, erm*(B), *aacA-aphD*	*hlgA/lukF/lukS, lukD/lukE, lukX/lukY*
Z121S	ST188	*M. nemestrina*	5/29/2015	Skin	t189	A	PEN, OXA, ERY, CLI, GEN, KAN, CIP	*blaZ, mecA, erm*(B), *aacA-aphD*	*hlgA/lukF/lukS, lukD/lukE, lukX/lukY*
Z123N1	ST188	*M. nemestrina*	5/15/2015	Nasal	t189	A	PEN, OXA, ERY, CLI, GEN, KAN, CIP	*blaZ, mecA, erm*(B), *aacA-aphD*	*hlgA/lukF/lukS, lukD/lukE, lukX/lukY*
Z123N2	ST188	*M. nemestrina*	5/29/2015	Nasal	t189	A	PEN, OXA, ERY, CLI, GEN, KAN, CIP	*blaZ, mecA, erm*(B), *aacA-aphD*	*hlgA/lukF/lukS, lukD/lukE, lukX/lukY*
Z123S	ST188	*M. nemestrina*	5/29/2015	Skin	t189	A	PEN, OXA, ERY, CLI, GEN, KAN, CIP	*blaZ, mecA, erm*(B), *aacA-aphD*	*hlgA/lukF/lukS, lukD/lukE, lukX/lukY*
Z1242N1	ST188	*M. nemestrina*	2/24/2015	Nasal	t189	A1	PEN, OXA, ERY, CLI, GEN, KAN, TET, CIP, BAC	*blaZ, mecA, erm*(B), *aacA-aphD, tet*(K), *qacC*	*hlgA/lukF/lukS, lukD/lukE, lukX/lukY*
Z1242N2	ST188	*M. nemestrina*	4/24/2015	Nasal	t189	A1	PEN, OXA, ERY, CLI, GEN, KAN, TET, CIP, BAC	*blaZ, mecA, erm*(B), *aacA-aphD, tet*(K), *qacC*	*hlgA/lukF/lukS, lukD/lukE, lukX/lukY*
Z1242N3	ST188	*M. nemestrina*	6/5/2015	Nasal	t189	A1	PEN, OXA, ERY, CLI, GEN, KAN, TET, CIP, BAC	*blaZ, mecA, erm*(B), *aacA-aphD, tet*(K), *qacC*	*hlgA/lukF/lukS, lukD/lukE, lukX/lukY*
Z130	ST188	*M. nemestrina*	2/25/2015	Nasal	t189	A1	PEN, OXA, ERY, CLI, GEN, KAN, TET, CIP, BAC	*blaZ, mecA, erm*(B), *aacA-aphD, tet*(K), *qacC*	*hlgA/lukF/lukS, lukD/lukE, lukX/lukY*
Z1304	ST188	*M. nemestrina*	7/2/2015	Nasal	t189	A	PEN, OXA, ERY, CLI, GEN, KAN, CIP	*blaZ, mecA, erm*(B), *aacA-aphD*	*hlgA/lukF/lukS, lukD/lukE, lukX/lukY*
Z131S	ST188	*M. nemestrina*	7/2/2015	Skin	t189	A	PEN, OXA, ERY, CLI, GEN, KAN, CIP	*blaZ, mecA, erm*(B), *aacA-aphD*	*hlgA/lukF/lukS, lukD/lukE, lukX/lukY*
Z143	ST188	*M. nemestrina*	2/18/2015	Nasal	t3887	A	PEN, OXA, ERY, CLI, GEN, KAN, CIP	*blaZ, mecA, erm*(B), *aacA-aphD*	*hlgA/lukF/lukS, lukD/lukE, lukX/lukY*
A140	ST 3268	*M. mulatta* WaNPRC SIV+	7/31/2015	Nasal	t15469	B	PEN, OXA, TET, CIP	*blaZ, mecA, tet*(K), *fosB*	*egc, hlgA/lukF/lukS, lukD/lukE, lukX/lukY sec, sel*
A1404N	ST 3268	*M. mulatta* WaNPRC SIV+	7/20/2015	Nasal	t15469	B	PEN, OXA, TET, CIP	*blaZ, mecA, tet*(K), *fosB*	*egc, hlgA/lukF/lukS, lukD/lukE, lukX/lukY sec, sel*
A1404W	ST 3268	*M. mulatta* WaNPRC SIV+	7/20/2015	Wound	t15469	B	PEN, OXA, TET, CIP	*blaZ, mecA, tet*(K), *fosB*	*egc, hlgA/lukF/lukS, lukD/lukE, lukX/lukY sec, sel*
A1408	ST 3268	*M. mulatta* Vendor #1	7/17/2015	Nasal	t15469	B	PEN, OXA, TET, CIP	*blaZ, mecA, tet*(K), *fosB*	*egc, hlgA/lukF/lukS, lukD/lukE, lukX/lukY sec, sel*
A1524	ST 3268	*M. fascicularis* China 6 mo quarantine in CA	4/8/2015	Nasal	t13638	B	PEN, OXA, (GEN), KAN, TET, CIP, BAC	*blaZ, mecA, aacA-aphD, tet*(K), *fosB, qacC*	*egc, hlgA/lukF/lukS, lukD/lukE, lukX/lukY sec, sel*
A1525	ST 3268	*M. fascicularis* Vendor #2 China	4/8/2015	Nasal	t13638	B	PEN, OXA, GEN, KAN, TET, CIP, BAC	*blaZ, mecA, aacA-aphD, tet*(K), *fosB, qacC*	*egc, hlgA/lukF/lukS, lukD/lukE, lukX/lukY sec, sel*
A109	ST 3268	*M. nemestrina* Indonesia 2010, arrived Seattle 2015	5/20/2015	Nasal	t13638	B1	PEN, OXA, GEN, KAN, TET, CIP	*blaZ, mecA, aacA-aphD, tet*(K), *fosB*	*egc, hlgA/lukF/lukS, lukD/lukE, lukX/lukY sec, sel*
K990W	ST 3268	*M. nemestrina* LA NPRC	8/5/2015	wound	t13638	B	PEN, OXA, (GEN), KAN, TET, CIP, BAC	*blaZ, mecA, aacA-aphD, tet*(K), *fosB, qacC*	*egc, hlgA/lukF/lukS, lukD/lukE, lukX/lukY sec, sel*
Z1403	ST 3268	*M. nemestrina* Born in TX arrived 2015	5/19/2015	Nasal	t13638	B2	PEN, OXA, (GEN), KAN, TET, CIP	*blaZ, mecA, aacA-aphD, tet*(K), *fosB*	*egc, hlgA/lukF/lukS, lukD/lukE, lukX/lukY sec, sel*


*All isolates carried the mecA gene. Pen, penicillin; Oxa, oxacillin; Cip, ciprofloxacin; Gen, gentamicin; Kan, kanamycin; Tet, tetracycline; BAC, benzalkonium chloride; brackets indicate an intermediate phenotype. All isolates were mupirocin susceptible and lacked the mupA gene. The enterotoxin H gene [entH], ORF CM14, and splE were absent in all isolates. The collagen-binding adhesin [cna] and the protease genes splA, splB were present in the ST188 isolates but were not detected among the ST3268 isolates. None of the 21 isolates carried PVL genes, the toxic shock syndrome toxin 1 gene [tst1], or the exfoliative toxin genes [etA, etB, etD], or genes associated with β-haemolysin converting phages (sea, see, scn, chp).*

Nine of the 21 ST3268 SCC*mec* V isolates were selected from animals representing different commercial vendors and out-state-location sources for the macaques. There were seven nasal samples and two wound samples. The isolates were selected without knowledge of the host primate species and included two isolates [A1404N nasal, A1404S skin sample both taken on July 20, 2015] from a SIV-positive *M. mulatta* [A1404] from WaNPRC. *M. mulatta* A1404 had close contact with the SIV-positive animal A140 [A140 nasal] and was also from the WaNPRC (**Table 1**). Both animals had a compromised immune system and bite wounds. The other six MRSA ST3268 isolates originated from macaques shipped from other United States primate sites, macaques shipped from two different commercial vendors [A1408, A1535] or directly shipped from China and having been in quarantine for 6 months in California before shipping to the WaNPRC [A1524] (**Table 1**). These nine isolates came from two *M. mulatta* [nasal isolates A140, A1404N, and one wound isolate A1404W], two *M. fascicularis* [nasal isolates A1524, A1525] and three *M. nemestrina* [two nasal A109, Z1403, one wound site isolate K990W] (**Table 1**).

All isolates were grown on Brucella agar (Difco Laboratories, Division BD Sparks, MD, United States) slants and shipped by courier to Germany for further molecular testing.

### DNA Microarray Analysis, MLST, SCC*mec* Typing and *spa* Typing

The Alere StaphyType^®^ DNA microarray was used for all isolates as previously described (Monecke et al., 2011, 2016). The microarray includes 334 target sequences and ∼170 separate genes and allelic variants including species markers, SCC*mec*, capsule, *agr* group typing markers, common antibiotic resistance genes, toxins and microbial surface components recognizing adhesive matrix molecules [MSCRAMM] genes. The latter genes comprise among others *clfA* and *clfB* (encoding clumping factors A and B), *fnbA* and *fnbB* (encoding fibronectin binding proteins A and B), *fib* (encoding fibrinogen binding protein), *eno* (encoding laminin binding protein), and *cna* (encoding collagen binding protein), the gene products of which play a role in the initial attachment of bacteria to host tissue. The detailed protocol as well as the sequences of primers and probes have previously been published (Monecke et al., 2011).

The clonal complexes (CCs) were determined by automated comparison of the microarray hybridization profiles to a database of previously characterized isolates (Monecke et al., 2011, 2016). The *spa* typing was performed according to Harmsen et al. (2003). The *spa* types were determined using the Ridom website.

The MLST typing was done using PCR and sequencing and the SCC*mec* typing was performed as previously described prior to being sent to Germany (Soge et al., 2016).

### Antimicrobial Susceptibility Testing

The antimicrobial susceptibility testing was performed for 30 antimicrobial agents by broth microdilution according to the Clinical and Laboratory Standards Institute (Clinical Laboratory Standard Institute [CLSI], 2018). The microtiter plates (MCS Swalmen, Netherlands) included penicillins (penicillin, ampicillin, amoxicillin/clavulanic acid, oxacillin), carbapenems (imipenem), a macrolide (erythromycin), a lincosamide (clindamycin), tetracyclines (tetracycline, doxycycline), aminoglycosides (gentamicin, streptomycin), a quinolone (ciprofloxacin), an oxazolidinone (linezolid), a glycopeptide (vancomycin), a streptogramin combination (quinupristin/dalfopristin), a phenicol (florfenicol), a pleuromutilin (tiamulin), and the combination trimethoprim/sulfamethoxazole. The aminoglycoside kanamycin was tested by broth macrodilution (Clinical Laboratory Standard Institute [CLSI], 2018, **Supplementary Table S1**). As there are no CLSI-approved clinical breakpoints applicable to primates other than humans, we used the human clinical breakpoints as listed in the CLSI document M100, 28th edition (Clinical Laboratory Standard Institute [CLSI], 2018). The breakpoints for the categories susceptible (S), intermediate (I) and resistant (R), are as follows: penicillin (S ≤ 0.12 μg/mL, R ≥ 0.25 μg/mL), oxacillin S ≤ 2 μg/mL, R ≥ 4 μg/mL, ciprofloxacin and quinupristin/dalfopristin (S ≤ 1 μg/mL, I = 2 mg/mL, R ≥ 4 μg/mL), gentamicin, doxycycline and tetracycline (S ≤ 4 μg/mL, I = 8 μg/mL, R ≥ 16 μg/mL), erythromycin (S ≤ 0,5 μg/mL, I = 1–4 μg/mL, R ≥ 8 μg/mL), clindamycin (S ≤ 0.5 μg/mL, I = 1–2 μg/mL, R ≥ 4 μg/mL), linezolid (S ≤ 4 μg/mL, R ≥ 8 μg/mL), trimethoprim/sulfamethoxazole (S ≤ 2/38 μg/mL, R ≥ 4/76 μg/mL), and vancomycin (S ≤ 2 μg/mL, I = 4–8 μg/mL, R ≥ 16 μg/mL) (Clinical Laboratory Standard Institute [CLSI], 2018, **Supplementary Table S1**). There are no clinical breakpoints for *S. aureus* for ampicillin, amoxicillin-clavulanic acid and imipenem, but if *S. aureus* strains are classified as resistant to oxacillin they are also considered as resistant to other β-lactams. Since there are no CLSI approved kanamycin breakpoints available, isolates with MICs of ≥ 64 μg/mL were tentatively considered as resistant (Feßler et al., 2010). Florfenicol and tiamulin are not used in human medicine and thus no breakpoints are available.

Susceptibility testing of the biocides benzalkonium chloride, chlorhexidine, glutardialdehyde, and isopropanol was also performed by broth macrodilution. For this, a bacterial suspension was prepared in a tryptone-saline-diluent (TSD; 1 g tryptone-peptone, 8.5 g sodium chloride in 1 L purified water) in a concentration of in 1 ×10^8^–1 × 10^9^ cfu/mL from 16 to 24 h old cultures on tryptic soy agar (TSA) (Roth, Karlsruhe, Germany). This suspension was diluted 1:10. From this dilution, 20 μl were added per each ml double concentrated tryptic soy broth (2× TSB) (Roth, Karlsruhe, Germany). One ml of this inoculum was added to a 2-fold benzalkonium chloride dilution series prepared in 1 mL volumes. The test ranges were as follows: benzalkonium chloride 0.00005–0.0008%, chlorhexidine 0.000025–0.0008%, glutardialdehyde 0.03–1%, and isopropanol 4 to 12%. The results were read after 24 h incubation at 37°C (Feßler et al., 2018).

### Macrorestriction Analysis With Subsequent Pulsed-Field Gel Electrophoresis (PFGE)

SmaI macrorestriction analysis with subsequent pulsed-field gel electrophoresis was performed as previously described (Murchan et al., 2003) and the gels were analyzed according to the criteria Tenover et al. (1995) and (Deng et al., 2017).

## Results

### Basic Characteristics of the ST188 SCC*mec* IV and ST3268 SCC*mec* V Isolates

Previously, nasal cultures were performed on 596 primates and 105 (17.6%) were MRSA positives. With the exception of four animals all in-house primates carried the MRSA ST188, while the MRSA ST3258 was associated with animals that were shipped into WaNPRC from other primate facilities and commercial breeders (Soge et al., 2016). *M. nemestrina* represent 75% of the primates in the WaNPRC. All ST188 and ST3268 isolates were positive for the species markers (*rrnD1, gapA, katA, coA, nuc1, spa, sbi*), capsule and *agr* alleles and consistent with an identification as *S. aureus*. All fifteen ST188 isolates selected for the study came from *M. nemestrina* hosts and were verified to have the ST188 MLST profile (3-1-1-8-1-1-1). All but one had *spa* type t189 (07-23-12-21-17-34), while the remaining isolate [Z143] had *spa* type t3887 (07-23-12-12-34). The nine ST3268 isolates had a MLST profile of 1-14-430-214-10-303-329. Two different *spa* types were identified, t13638 (*n* = 5) and t15469 (*n* = 4). The two *spa* types differed by the presence of an additional repeat 17 in *spa* type t15469 (210-23-02-34-17-34-34-17-17-23-34) compared to *spa* type t13638 (210-23-02-34-17-34-34-17-23-34) (**Table 1**). The *spa* type t13638 isolates were cultured from *M. fascicularis* and *M. nemestrina*. This *spa* type was first described in a methicillin-susceptible *S. aureus* from the United Kingdom^2^. The *spa* type t15469, cultured from *M. mulatta*, is a novel *spa* type, first described in these primate isolates^2^ (**Table 1**). In the ST3268 isolates, the *spa* types correlated with the host macaque species. The four isolates from *M. mulatta* hosts were *spa* type t15469, while the two *M. fascicularis* and *M. nemestrina* isolates were *spa* type t13638 (**Table 1**).

### PFGE Profiles

Nine of 15 ST188 [L091 (nasal), Z121N (nasal), Z121S (skin), Z123N1 (nasal) and Z123N2 (nasal), Z123S (skin), Z1304 (nasal), Z131S (skin), and Z143 (nasal)], from six *M. nemestrina* had indistinguishable PFGE patterns [A]. Five ST188 isolates originating from three animals [K062 (nasal), Z1242N1, Z1242N2 and Z1242N3 (nasal), and Z130 (nasal) shared PFGE sub-pattern [A1], while the ST188 isolate A112 (nasal) had a second PFGE sub-pattern [A2] (**Table 1** and **Supplementary Figure S1**).

Of the nine MRSA ST3268 isolates, seven [A140 (nasal), A1404N (nasal), A1404W (wound), A1408 (nasal), A1524 (nasal), A1525 (nasal), and K990W (wound)] had the same PFGE pattern [B]. The isolates A1404N (nasal) and A1404W (wound) were cultured from the same animal eleven days apart and were indistinguishable in their PFGE patterns, their resistance pheno- and genotypes, as well as, their virulence genes (**Table 1**). The two sub-patterns B1 and B2 were found in found in single isolates A109 (nasal) and Z1403 (nasal), respectively (**Table 1** and **Supplementary Figure S1**).

### Resistance Pheno- and Genotypes of the ST188 SCC*mec* IV and ST3268 SCC*mec* V Isolates

All 21 MRSA isolates were resistant to penicillin and oxacillin. They carried the *mecA* gene and the β-lactamase gene *blaZ.* All isolates were also resistant to ciprofloxacin. In addition, all ST188 isolates were resistant to macrolides and lincosamides via the *erm*(B) gene and carried the aminoglycoside resistance gene *aacA-aphD* mediating gentamicin and kanamycin resistance. The *aacA-aphD* gene was only present in five of the ST3268 isolates, which exhibited high kanamycin MICs (≥ 256 mg/L) and were classified as resistant or intermediate to gentamicin. The nine MRSA ST3268 isolates were all tetracycline resistant and carried the *tet*(K) gene, while only five MRSA ST188 isolates (K062, Z1242N1, Z1242N2, Z1242N3, and Z130), from 3 *M. nemestrina*, were resistant to tetracycline and carried the *tet*(K) gene (**Table 1**).

From some of the animals, several isolates taken at different time points [Z1242N2, Z1242N3, Z123N1, and Z123N2] were included. However, even after one or more rounds of mupirocin topical treatment and chlorhexidine baths, the MRSA isolates either persisted in the noses of these juvenile animals or the animals were re-infected or re-colonized. Treatment success was measured by MRSA-negative cultures at two and four weeks after treatment. If the animal was still MRSA-positive, it was considered as treatment failure. If this happened, the animal was retreated with mupirocin and chlorhexidine baths. This primarily happened in juvenile animals. Because this “treatment failure” was limited to juvenile animals the veterinarian staff felt that it suggested that the animals were refractory to clearance of the isolate, the isolate may have become resistant to mupirocin due to acquisition of the mupirocin gene *mupA* or an alternative resistance mechanism, or other characteristic of being a juvenile *M. nemestrina* rather than clearance and reinfection since there was no sign of clearance in two and four week samples (**Table 1**). However, none of these isolates or any of the other isolates in the study were resistant to mupirocin nor did they carry the *mupA* gene (**Table 1**).

All the isolates were tested for reduced susceptibility to benzalkonium chloride, while no change was seen with chlorhexidine, glutardialdehyde, or isopropanol. Some isolates including ST188 isolates K062, Z1242N1, Z1242N2, Z1242N3, Z130, and ST3268 isolates A1524, A1525, and K990W, had a benzalkonium chloride MIC of 0.0004% and carried the *qacC* gene. All other isolates, that did not harbor the *qacC* gene, had benzalkonium chloride MICs of 0.0001% (**Table 1**). No other change in the MIC of disinfectants were observed.

### Characterization of Accessory and Virulence Genes

The nine ST3268 isolates had the enterotoxin gene cluster *egc* [*seg, sei, selm, seln, selo, selu*] and the additional enterotoxin genes *sec* and *sel*. In contrast, none of the ST188 harbored the enterotoxin gene cluster *egc, sec* or *sel* genes (**Table 1**). The fifteen ST188 and nine ST3268 isolates carried the *hlgA* locus [comprising of *hlgA/lukF/lukS*], leukocidin genes [*lukD/E* and *lukX/Y*], the aureolysin gene [*aur*], and the protease genes *sspA, sspB*, and *sspP*. The gene for the *S. aureus* surface protein G [*sasG*] was present among the ST3268 isolates but absent in the ST188 isolates. Two isolates were additionally tested with a new array and both A1403 and Z140 were positive for the carotinoid pigment gene cluster [*crtM/N/O/P*]. Other isolates were not tested.

In contrast, the enterotoxin H gene [*entH*], ORF CM14, and *splE* were absent in all isolates (**Table 1**). The collagen-binding adhesin [*cna*] and the protease genes *splA, splB* were present in the ST188 isolates but were not detected among the ST3268 isolates. None of the 21 isolates carried PVL genes, the toxic shock syndrome toxin 1 gene [*tst1*], exfoliative toxin genes [*etA, etB, etD*], or genes associated with β-haemolysin converting phages (*sea, see, scn, chp*) (**Table 1**).

Two ST3268 SCC*mec* V isolates, A140 from a *M. mulatta* and Z1403 from a *M. nemestrina*, were further tested for SCC*mec* accessory genes. The following genes were identified in both *mvaS, cstB-SCC2, ydhK, D1GU38, Q4LAG7, czrC, “ccrAA”* (a recombinase homologue associated with *ccrC*), and a SCC*mec* terminus type 2 (Monecke et al., 2016). This is consistent with the presence of SCC*mec* VT+*czrC* composite elements as described for the CC398 strain SO385 (GenBank accession number AM990992.1), a livestock-associated MRSA strain from Western Europe (Schijffelen et al., 2011).

All isolates from the same animal shared indistinguishable PFGE patterns, regardless of whether nasal samples were taken at different times, or nasal and skin samples taken at the same time from the same *M. nemestrina*. As shown below, isolates from the same animal were also indistinguishable with respect to their resistance pheno- and genotypes, and other genes including enterotoxin, hemolysin, leukocidin, or PVL genes (**Table 1**), suggesting the presence of the same or a closely related strain in different locations of the animal and/or the persistence of that strain over time.

## Discussion

There have been two different clones present in macaques from the WaNPRC facility. The in-house clone ST188 was primarily found in *M. nemestrina*, the predominant primate species [75% of the primates] in the WaNPRC facility. At this time, we believe it was introduced into the facility from primates shipped from other United States National Primate Research Facility and/or commercial vendors around 2014. Then this clone was spread across the facility mainly via in-house transmission. ST188 has continued to be isolated from primates in 2018 and from the primate environment in 2018.

The second MRSA clone ST3268 came from primates that were originally shipped from two different commercial breeders in two different states and other primate colonies in the United States. ST3268 was identified for the first time after a United States facility shipped ∼90 animals in May 2015 to the WaNPRC. In 2016, MRSA ST3268 SCC*mec* V-positive animals were also shipped from a third commercial vendor in a third state to WaNPRC suggesting that this is the primary way ST3268 has continued to be introduced into the WaNPRC. The vendor animals primarily originated from China or Indonesia. The four MRSA ST3268-positive WaNPRC animals were those that had contact with MRSA ST3268-positive animals by following them into a treatment room. Hence, the assumption was that the treatment room was contaminated with MRSA ST3268 and the SIV-positive animals picked up the strain in the treatment room. Similarly, we have found ST188-positive macaques from both commercial vendors and other United States primate facilities. The original source of the MRSA ST188 is not as clear though it can be found in low prevalence among humans in Asia (Soge et al., 2016).

As previously reported (Soge et al., 2016) MRSA ST188 isolates have been isolated almost exclusively from Asian humans but these strains often carry other SCC*mec* types then found in the WaNPRC primates. This MLST type is very rarely reported in North America. One report has identified MSSA ST188 from sanctuary chimpanzees isolated in Uganda and ten MSSA ST188 isolated from wild Madagascar lemurs. The major differences between the two clones other than MLST and *spa* type is that ST188 has primarily been associated with *M. nemestrina*, the predominate primate in WaNPRC. In contrast, ST3268 has been identified in all three species of macaques in the WaNPRC. The two clones also differ in the carriage of antimicrobial resistance genes. For example, the *erm*(B) gene is present in all ST188 isolates studied; but none of the ST3268 isolates in the current study harbored this gene. The *tet*(K) gene is present in all ST3268 in the current study, but only in some of the ST188 isolates (**Table 1**). Only ST3268 isolates carried the *fosB* gene. All isolates from both clones were ciprofloxacin resistant. The mechanism of resistance to ciprofloxacin was not determined, however, in our previous study with related isolates from macaques in the WaNPRC center both ST188 and ST3268 isolates carried a *gyrA* mutation that resulted in the Ser84Leu amino acid substitution, suggesting that the isolates in the current study may also have this mutation (Soge et al., 2016). A few isolates of both clones had elevated benzalkonium chloride MICs.

For other genes, there were differences in the carriage of the *egc* gene cluster, *sec* and *sel* genes with all ST3268 isolates and none of the ST188 isolates carrying these genes. However, none of the differences in genes identified could readily explain the different ability to transfer between the primates within the WaNPRC or the lack of finding ST3268 in environmental sites both in 2015 and more recently in 2018 (data not shown). Recently, Hsu et al. (2017) identified six ST3268 SCC*mec* V and two ST2817 SCC*mec* isolates taken from *M. fascicularis* used in experimental surgery in 2014 in Singapore. An additional isolate was cultured from a person who worked in animal husbandry in the facility. These animals primarily came from Vietnam and were imported between 2009 and 2014. Both MLST types can be regarded as belonging to the same clonal complex (Hsu et al., 2017). The Singaporean ST3268 SCC*mec* V isolates were resistant to ciprofloxacin, gentamicin and tetracycline. MICs were determined but specific antibiotic resistance genes were not identified in the Hsu et al., 2017. One Singapore isolate, DN260, differed from ST3268 WaNPRC United States, TXA, and TXB isolates by 36 SNPs (Soge et al., 2016; Hsu et al., 2017). It was unclear in the Hsu study whether the ST3268 was able to transfer between animals within their facility or if they came into the facility carrying the MRSA. However, it is possible that the facility worker acquired his nasal MRSA ST3268 from the MRSA-positive primates or contaminated work environment.

ST3268 is genetically related to ST2817 which is found in low prevalence in Asia, previously isolated from a human surgical wound in Singapore in 2014^3^. However, except for the one worker all MRSA ST3268 SCC*mec* V isolates have been isolated from macaques and thus may very well be a primate-associated strain that is common in parts of Asia (Soge et al., 2016; Hsu et al., 2017).

The ST188 clone continues to be the dominant MRSA clone in the WaNPRC. We examined the two MRSA isolates recovered in Aug 2017 and both were ST188. As previously shown, we also found a few methicillin susceptible *S. aureus* [MSSA] strains that were ST188 which clustered with the MRSA ST188 from the WaNPRC primates (Soge et al., 2016). No MSSA that were ST3268 have been identified though the number of MSSA examined has been small (Soge et al., 2016). The MRSA ST3268 isolates characterized in the current publication were recovered over a seven month time period, and could be subdivided into two *spa* types, which were found in different species of macaques (**Table 1**).

The data from the current study as well as previous studies (Soge et al., 2016; Hsu et al., 2017) suggest that all primates should be screened and treated for MRSA carriage prior to being shipped to other facilities within a country or between countries to reduce the continual spread of primate-related MRSA.

## Conclusion

The primate isolates belonged to two different clones, ST188 and ST3268. ST188 was the in-house clone that easily spread among primates in the colony. It was primarily identified in *M. nemestrina*, though this could be due to the predominance [75%] of this species of macaques in the WaNPRC. Fourteen of the 15 ST188 isolates exhibited the same *spa* type t189. Five isolates carried the *tet*(K) gene coding for tetracycline resistance and all had PFGE pattern A1 with all five of these isolates harboring the *qacC* gene and showing reduced susceptibility to benzalkonium chloride. The nine ST188 isolates with PFGE pattern A were susceptible to tetracyclines and did not carry tetracycline resistance genes. The other clone, ST3268, was introduced from external macaques shipped from other United States primate facilities and United States commercial companies. ST3268 did not spread easily among the primates even though each isolate carried the *egc* enterotoxin gene cluster, *sec* and *sel* genes. One unexpected observation with the ST3268 isolates was finding that the *spa* type varied by macaque host species as did the mobile antibiotic resistance genes and reduced susceptibility to benzalkonium chloride. However, seven out of nine isolates had the same PFGE pattern B and the two variants PFGE patterns B1 and B2 did not correlate with either host macaque species or antibiotic resistance genes carried suggesting that they are members of a closely related clone. The data presented does not provide insight into why ST188 could spread easily while ST3268 did not spread within the WaNPRC facility.

## Ethics Statement

Primate samples were taken as part of the general care of the animals.

## Author Contributions

MR designed the experiments. DN did the laboratory work in Seattle. AF and SS did the laboratory work in Germany. SM and RE helped us to understand the results. All authors worked on writing the manuscript up for publications.

## Conflict of Interest Statement

The authors declare that the research was conducted in the absence of any commercial or financial relationships that could be construed as a potential conflict of interest.

## Footnotes

1 https://pubmlst.org/saureus/

2 http://spa.ridom.de/

3 http://saureus.mlst.net/
