# Supplementary material for: Molecular Analysis of Two Different MRSA Clones ST188 and ST3268 From Primates (Macaca spp.) in a United States Primate Center
Source: Front Microbiol. 2018 Oct 9;9:2199. doi: 10.3389/fmicb.2018.02199 (PMC6190752; doi:10.3389/fmicb.2018.02199)
Supplement: Supplementary file 1 [file Data_Sheet_1.doc]

Figure 1. SmaI PFGE patterns A [isolate # L091], A1 [isolate # K062], A2 [isolate # A112], B [isolate # A140], B1 [isolate # A109], and B2 [isolate # Z1403 ], obtained among the ST3268 MRSA isolates. M=marker (SmaI digested *S. aureus* NCTC 8325). Cluster analysis was performed with BioNumerics 7.5 using the band-based method selecting the parameters Dice (band matching tolerance 1%) and UPGMA.
