# Supplementary material for: Molecular Analysis of Two Different MRSA Clones ST188 and ST3268 From Primates (Macaca spp.) in a United States Primate Center
Source: Front Microbiol. 2018 Oct 9;9:2199. doi: 10.3389/fmicb.2018.02199 (PMC6190752; doi:10.3389/fmicb.2018.02199)
Supplement: Supplementary file 2 [file Table_1.DOC]

Table S1. Test ranges of the antimicrobial agents

| **Antimicrobial agents** | **Test ranges**  **(in µg/mL)** | **CLSI breakpoints (M100 28^th^ ed) (in µg/mL)** | | |
| --- | --- | --- | --- | --- |
|  |  | **susceptible** | **intermediate** | **resistant** |
| penicillin | 0.015 - 32 | ≤ 012 | - | ≥ 0.25 |
| ampicillin | 0.03 - 64 |  |  |  |
| amoxicillin/clavulanic acid | 0.03/0.015 - 63/32 |  |  |  |
| oxacillin | 0.015 - 8 | ≤ 2 | - | ≥ 4 |
| imipenem | 0.015 - 32 |  |  |  |
| erythromycin | 0.015 - 32 | ≤ 0.5 | 1-4 | ≥ 8 |
| clindamycin | 0.03 - 64 | ≤ 0.5 | 1-2 | ≥ 4 |
| tetracycline | 0.12 - 256 | ≤ 4 | 8 | ≥ 16 |
| doxycycline | 0.06 - 128 | ≤ 4 | 8 | ≥ 16 |
| gentamicin | 0.12 - 256 | ≤ 4 | 8 | ≥ 16 |
| streptomycin | 0.25 - 512 |  |  |  |
| ciprofloxacin | 0.008 - 16 | ≤ 1 | 2 | ≥ 4 |
| linezolid | 0.03 - 64 | ≤ 4 | - | ≥ 8 |
| vancomycin | 0.015 - 32 | ≤ 2 | 4-8 | ≥ 16 |
| quinupristin/dalfopristin | 0.015 - 32 | ≤ 1 | 2 | ≥ 4 |
| florfenicol | 0.12 - 256 |  |  |  |
| tiamulin | 0.03 - 64 |  |  |  |
| trimethoprim/sulfamethoxazol | 0.015/0.3 - 32/608 | ≤ 2/38 | - | ≥ 4/76 |
| kanamycin | 0.5 - 256 |  |  |  |
